# Metabolome and transcriptome profiles in quinoa seedlings in response to potassium supply

**DOI:** 10.1186/s12870-022-03928-8

**Published:** 2022-12-21

**Authors:** Tingzhi Huang, Xuesong Zhang, Qianchao Wang, Yirui Guo, Heng Xie, Li Li, Ping Zhang, Junna Liu, Peng Qin

**Affiliations:** Yunnan Agricultural University, Panlong District, Yunnan Province Kunming City, China

**Keywords:** Quinoa, Potassium, Metabolome, Transcriptome, Photosynthesis, Plant growth, Arginine biosynthesis

## Abstract

**Background:**

Quinoa (*Chenopodium quinoa* Willd.) is a herb within the Quinoa subfamily of Amaranthaceae, with remarkable environmental adaptability. Its edible young leaves and grains are rich in protein, amino acids, microorganisms, and minerals. Although assessing the effects of fertilization on quinoa yield and quality has become an intensive area of research focus, the associated underlying mechanisms remain unclear. As one of the three macro nutrients in plants, potassium has an important impact on plant growth and development. In this study, extensive metabolome and transcriptome analyses were conducted in quinoa seedlings 30 days after fertilizer application to characterize the growth response mechanism to potassium.

**Results:**

The differential metabolites and genes present in the seedlings of white and red quinoa cultivars were significantly enriched in the photosynthetic pathway. Moreover, the PsbQ enzyme on photosystem II and delta enzyme on ATP synthase were significantly down regulated in quinoa seedlings under potassium deficiency. Additionally, the differential metabolites and genes of red quinoa seedlings were significantly enriched in the arginine biosynthetic pathway.

**Conclusions:**

These findings provide a more thorough understanding of the molecular changes in quinoa seedlings that occur under deficient, relative to normal, potassium levels. Furthermore, this study provides a theoretical basis regarding the importance of potassium fertilizers, as well as their efficient utilization by growing quinoa seedlings.

**Supplementary Information:**

The online version contains supplementary material available at 10.1186/s12870-022-03928-8.

## Introduction

*Chenopodium quinoa* Willd, also known as quinoa, South American quinoa, and Indian wheat, among other names, is a dicotyledonous plant that belongs to the subfamily Quinoa of the family Amaranthaceae. According to Food and Agriculture Organization, quinoa has rich and uniform nutritional value and is capable of meeting many of the nutritional needs of humans. Additionally, it has been described as an excellent “functional food” with the capacity to reduce the risk of various diseases [1–3]. Quinoa is not only rich in macronutrients, such as high-quality proteins, polysaccharides, and unsaturated fatty acids, but also contains high levels of micronutrients, including vitamins and minerals. In addition, quinoa has higher contents of riboflavin, folic acid, calcium, magnesium, iron, and zinc than other grains. It also contains various phytochemicals, including, saponins, polyphenols, flavonoids, betaine, and phytosterols [4–6]. Concomitantly, quinoa is a plant with a high level of resistance to cold, salt, and drought, that grows well under different adverse climatic conditions [7].

Given that potassium (K) is present in nearly 5% of all higher plant tissues, it is considered an essential macronutrient. Although it is not a structural component of organic compounds, it is closely related to plant growth, development, and metabolism, with a particularly important impact on plant yield, quality, and stress resistance. For instance, K is a necessary component of the stomatal opening mechanism and, therefore, directly impacts stomatal conductance and photosynthesis, phloem transport, and cell ion balance. Moreover, K can promote chlorophyll synthesis in leaves, thereby improving the structure of chloroplasts and promoting plant photosynthesis, while also promoting the transportation and storage of various nutrients, increasing the sugar and starch contents in the crop, and promoting oil formation [8]. Importantly, K also functions in the assimilation of soluble proteins, carbohydrates, and nitrogen compounds formed in plant cells, while contributing to the mechanisms associated with countering heavy metal toxicity and disease tolerance [9, 10].

Under an unlimited K supply, the transport rate in plant vascular tissues is increased, plant metabolism is promoted, and plant resistance to lodging, disease and insect attack, and drought and cold are effectively enhanced [2, 11–14]. Conversely, under an insufficient K supply, stems grow weak and become lodging-prone, leaves readily lose water, and drought and cold tolerance decreases significantly. Furthermore, proteins and chlorophyll are degraded causing leaves to first turn chlorotic and then necrotic [15]. Hence, within a certain range, crop yield increases with increasing K fertilizer application rate; however, an excess of potassium fertilizer decreases crop yield.

Numerous studies have characterized the relationships between fertilizers and metabolism, including the responses of maize and barley to low nitrogen stress [16, 17]; common beans and barley to phosphorus deficiency [18, 19]; and Arabidopsis to sulfur deficiency [20], and the metabolite spectra of Arabidopsis and tomato plants under normal and low K conditions [21, 22]. Additionally, K significantly impacts the complex plant mechanisms [23, 24] underlying plant homeostasis [25], metabolic control [26], photosynthesis [27], and growth [28] in response to different stress conditions. Meanwhile, few studies have examined the plant mechanisms in response to K fertilizer application using a combined metabolome and transcriptome approach.

In this study, we aimed to determine the effect of K fertilizer rate on metabolome and transcriptome profiles of quinoa and analyze the response mechanism of quinoa seedlings to K fertilizer application through correlation analysis. To avoid one-sided results of a single cultivar, two quinoa cultivars, namely, red (dianli-1299) and white (dianli-71), were analyzed in this study. Each cultivar was treated under three conditions with a K_2_O gradient of 0 (R6, W6), 112.5 (R2, W2), or 337.5 kg/hm^2^ (R7, W7). R2 and W2 represented the control treatments. Collectively, our findings provide theoretical support for the research of high-yield cultivation technology for quinoa.

## Results

### Quality control of metabolome data

**Fig. 1:**
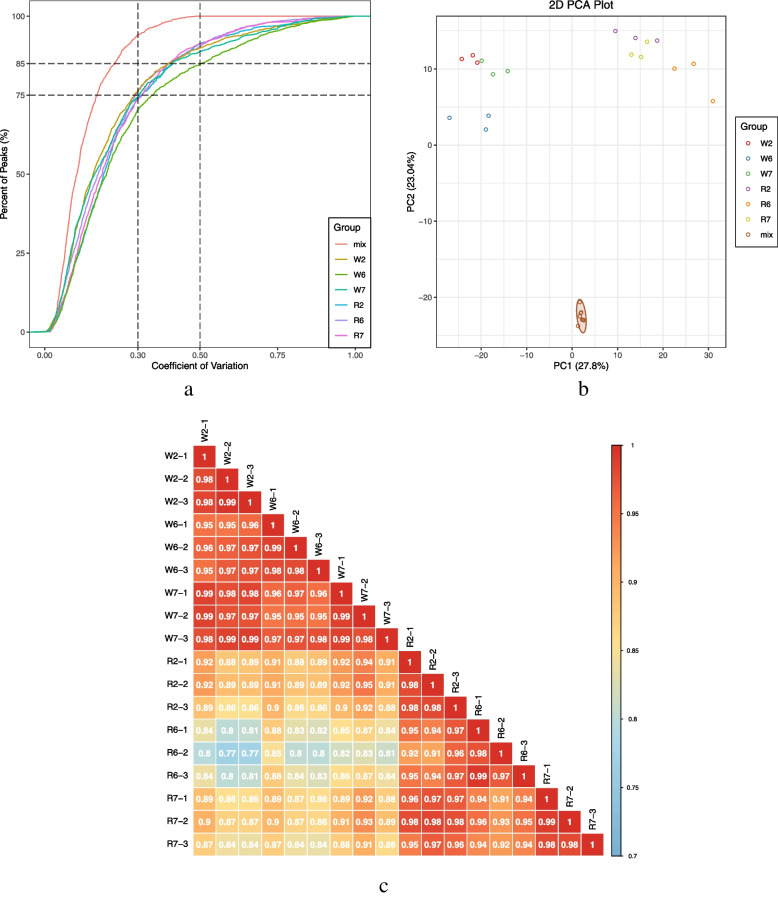
**a**) CV distribution of samples in each group; **b**) PCA score chart of mass spectrum data for each group of samples and quality control samples; **c**) diagram of correlation between samples

The superposition diagram (Fig. S1a.b) for the quality control (QC) sample generated via mass-spectrometry detection, as well as the total ion-flow diagram (total ion current/TIC) revealed a high level of overlap in the metabolite detection of total ion flow as well as consistent retention time and peak intensity, indicating that when mass spectrometry detected the same sample at different times, the signal stability was adequate, thus ensuring data repeatability and reliability. The proportion of substances with coefficient of variation (CV) values < 0.5 in QC samples was > 85%, whereas the proportion of substances with CV values < 0.3 was > 75%, except for W6, indicating that the W6 experimental data were relatively stable, while those of other groups were highly stable (Fig. 1a). Moreover, based on the principal component analysis (PCA) diagram (Fig. 1b), the data for each group showed appropriate repeatability and stability. This was confirmed by the correlation analysis between samples (Fig. 1c), which exhibited high repeatability of each treatment and high correlation between samples.

### Quantitative analysis of metabolome profiles

Sample metabolites were qualitatively and quantitatively detected using extensive targeted metabolome analysis. Eighteen samples were divided into six groups; biological triplicates were included for each sample. A total of 1057 metabolites were detected based on the Ultra high performance liquid chromatography tandem mass spectrometry (UPLC-MS/MS) detection platform and a self-built database. Among them were 98 amino acids and their derivatives, 166 phenolic acids, 69 nucleotides and their derivatives, 172 flavonoids, 24 lignans and coumarins, 9 tannins, 78 alkaloids, 39 terpenoids, 94 organic acids, 177 lipids, 11 quinones, and 120 other metabolites (Table S1).

**Fig. 2:**
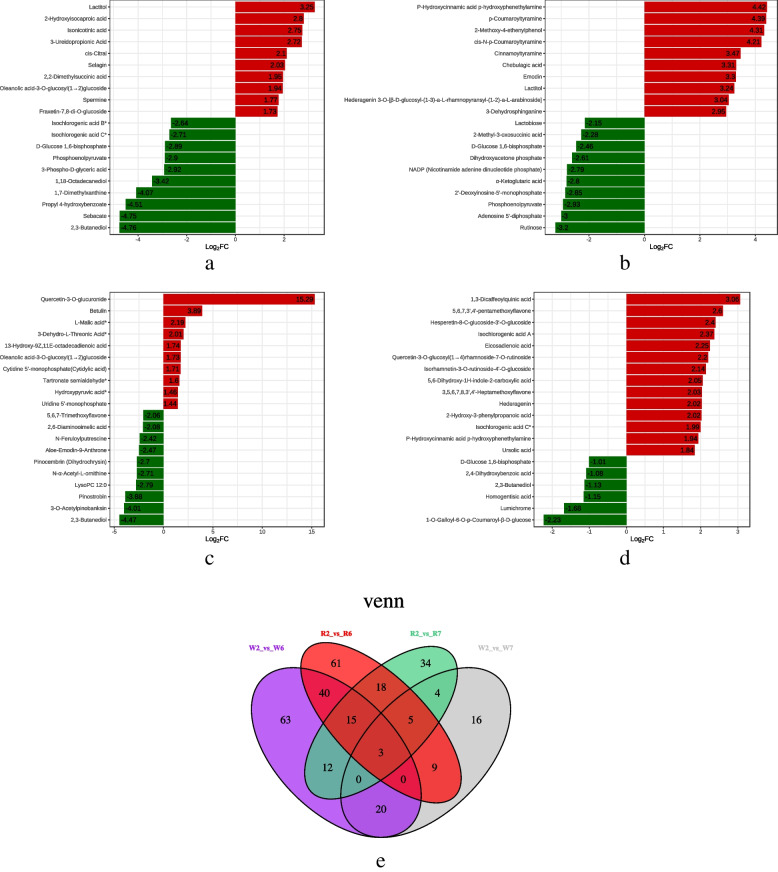
Column diagram of differential metabolites. **a**) R2 vs R6; **b**) W2 vs W6; **c**) R2 vs R7; **d**) W2vsW7. Venn diagram. **e**) W2 vs W6; W2 vs W7; R2 vs R6; and R2 vs R7

According to the classification of differentially expressed metabolites (DEMs), the top 10 DEMs in R2 vs R6 primarily included organic acids. The top included four organic acids; additionally, three of the top 10 downregulated metabolites were phenolic acids, three were sugars and alcohols, two were organic acids, and one corresponded to lipids, nucleotides, and their derivatives (Fig. 2a). Meanwhile, the top 10 upregulated metabolites DEMs in W2 vs W6 primarily included phenolic acids and alkaloids, with four sugars and alcohols, three organic acids, and three nucleotides and their derivatives (Fig. 2b). Four of the top 10 upregulated metabolites in the R2 vs R7 comparison were organic acids, with quercetin-3-o-glucuronide—a flavonol—found to be the most differentially abundant metabolite. In contrast, the top 10 downregulated DEMs in this comparison primarily included flavonoids (Fig. 2c). Lastly, the top 14 DEMs in W2 vs W7 primarily included phenolic acids (5/14) and flavonoids (5/14), with the top six found to primarily comprise phenolic acids, sugars, and alcohols (Fig. 2d).

According to the Venn diagram constructed for the four comparisons (Fig. 2e), 58 common DEMs were detected in W2 vs W6 and R2 vs R6, 12 in W2 vs W7 and R2 vs R7, and 3 in R2 vs R6, R2 vs R7, W2 vs W6 and W2 vs W7, namely, α-acetyl-l-ornithine, d-glucose-1,6-diphosphate, and isochlorogenic acid B *.

**Fig. 3:**
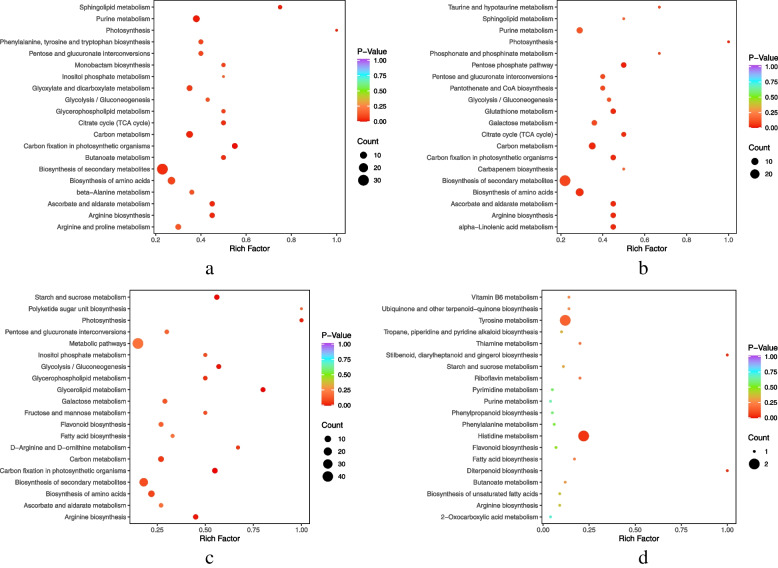
Enrichment analysis of differential metabolic pathways. **a**) R2 vs R6; **b**) W2 vs W6; **c**) R2 vs R7; and **d**) W2 vs W7

The primary differential metabolic pathways were selected via integration of differential metabolite pathways (Fig. 3). Among them, 151 DEMs were identified in the R2 vs R6 comparison, 78 of which were upregulated and 73 downregulated; 153 DEMs were detected in the W2 vs W6 comparison, among which 113 were upregulated and 40 were downregulated. Moreover, the differentially expressed metabolic pathways in the R2 vs R6 and W2 vs W6 comparisons were largely enriched in “carbon fixation in photosynthetic organisms,” “carbon metabolism,” “arginine biosynthesis,” “ascorbate and aldarate metabolism,” and “photosynthesis and citrate (TCA) cycle.”

Additionally, 91 DEMs were identified in the R2 vs R7 comparison, including 32 upregulated and 59 downregulated DEMs, while 57 DEMs were identified in the W2 vs W7 comparison, including 51 upregulated and 6 downregulated. However, there was no significant difference in metabolic pathways between R2 vs R7 or W2 vs W7. Nevertheless, R2 vs R6, R2 vs R7, and R6 vs R7 shared a common metabolic pathway, namely, arginine biosynthesis.

### Gene expression quantification and annotation

**Fig. 4:**
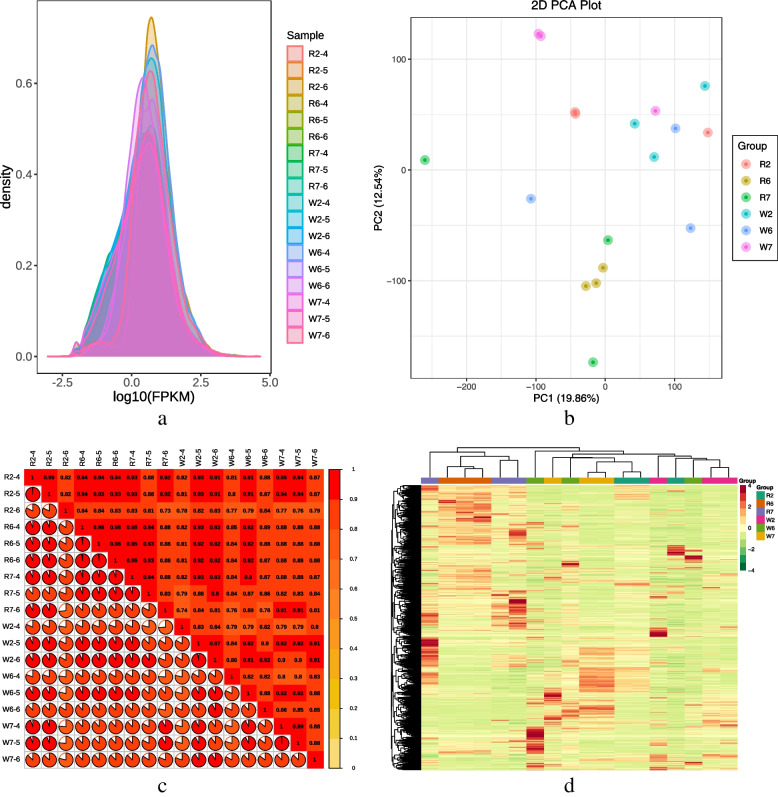
**a**) Gene expression density map; **b**) PCA score chart; **c**) Sample correlation diagram; **d**) Cluster heat map

Transcriptome sequencing in seedlings of the two quinoa cultivars was analyzed. After filtering the original data and checking the sequencing error rate and GC content distribution, 156.16 GB of clean data were obtained from 18 samples. The clean data of each sample reached 6 GB, and the percentage of Q30 base was ≥ 91%. The comparison of clean reads after QC to the reference genome showed a comparison efficiency of > 90%, indicating that the sequencing results satisfied the needs of subsequent analysis. The gene expression density map showed the gene abundance and expression trends in the 18 samples, which clearly indicated that the gene expression (Fragments Per Kilobase of transcript per Million fragments mapped, FPKM) in the samples was concentrated in log10 ^−2.5^-log10 ^2.5^ (Fig. 4a). Furthermore, based on the PCA score diagram (Fig. 4b), sample correlation diagram (Fig. 4c), and cluster heat diagram (Fig. 4d), the biological repeatability of the quinoa grain samples of the two cultivars were high, with differences detected between them. The genes detected in this experiment were annotated in the following databases: NR (53,275 genes), Swiss-Prot (34,131 genes), GO (41,046 genes), TrEMBL (51,060 genes), KOG (48,791 genes), Pfam (42,701 genes), and KEGG (29,232 genes).

### Differentially expressed gene-screening between seedlings of two quinoa cultivars growing under different potassium treatments

**Fig. 5:**
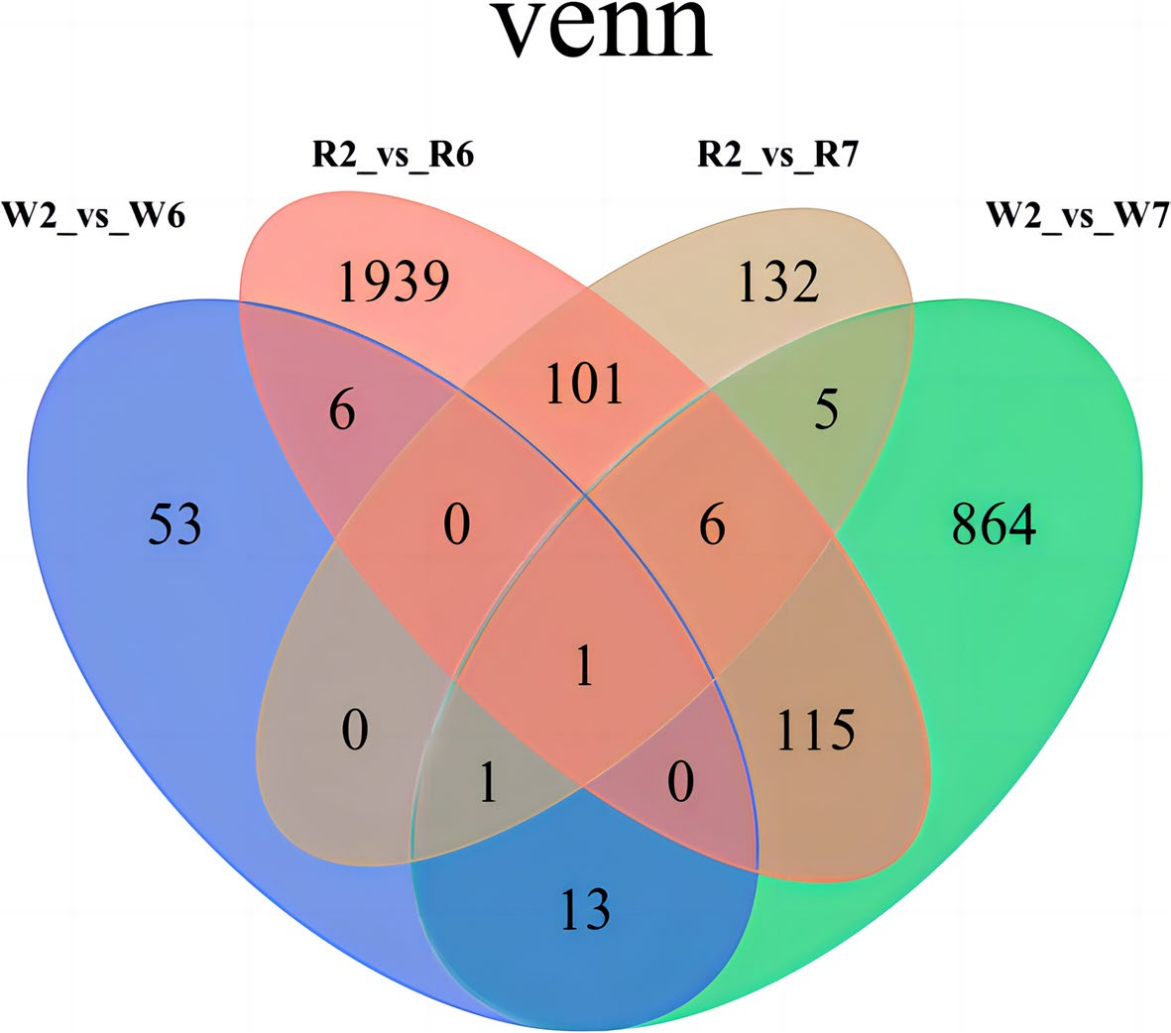
Differentially expressed gene-screening between seedlings of two quinoa cultivars growing under different potassium treatments

To assess gene expression patterns under different K supply levels, we first centralized and standardized the FPKM of genes and then performed K-Means cluster analysis. Considering that the same types of genes exhibited similar expression trends under different experimental treatments, we hypothesized that they might share similar functions (Fig. S2). According to the K-Means clustering diagram, differentially expressed genes (DEGs) were divided into 12 clusters, among which, the overall trend in expression change was similar between clusters 3, 6, 8, 9, and 11 across treatments; thus, DEGs were applied to compare potential DEGs in quinoa plants growing under different K fertilizer application rates. The Venn diagram (Fig. 5) shows that R2 vs R6 and W2 vs R6 shared seven DEGs, namely, gene-loc110710547, gene-loc110688217, gene-loc110705372, gene-loc110717944, gene-loc110719473, gene-loc110720427, and novel 559. Similarly, there were 13 common DEGs between R2 vs R7 and W2 vs W7, namely, gene-loc110684199, gene-loc110686087, gene-loc110688533, gene-loc110693212, gene-loc110694729, gene-loc110699212, gene-loc110699626, gene-loc110705642, gene-loc110707380, gene-loc110710547, gene-loc110720399, gene-loc110721931, and gene-loc110730331.

### Enrichment and screening of differentially expressed genes

**Fig. 6:**
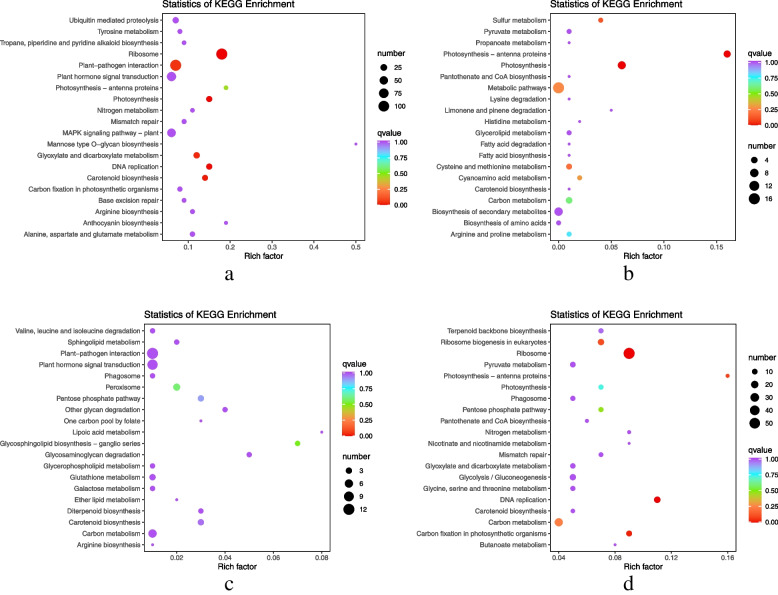
KEGG enrichment analysis of DEGs. **a**) R2 vs R6; **b**) W2 vs W6; **c**) R2 vs R7; **d**) W2 vs W7

The results of KEGG (Kyoto Encyclopedia of Genes and Genomes) annotation of DEGs revealed that the DEGs of each group were primarily enriched in “cellular process,” “environmental information process,” and “genetic information process.” Moreover, the common DEGs between R2 vs R6 and W2 vs W6 were mainly enriched in “photosynthesis,” “photosynthetic antenna protein,” “arginine biosynthesis,” and “carbon metabolism pathway.” Meanwhile, the common DEGs between R2 vs R7 and W2 vs W7 were largely enriched in “pentose phosphate pathway” (Fig. 6).

### Integration analysis of differentially expressed genes and differentially expressed metabolites through KEGG analysis

**Fig. 7:**
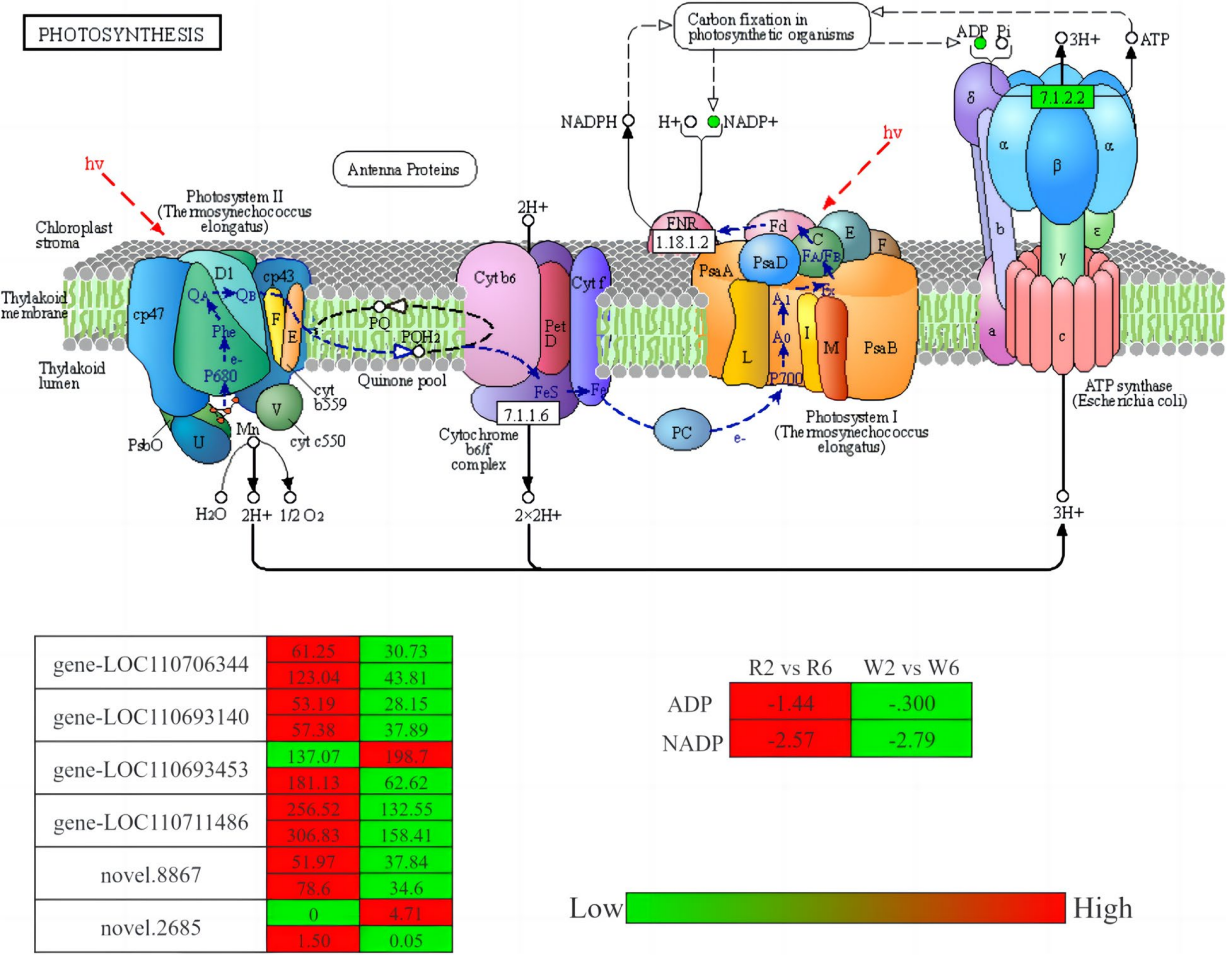
Photosynthetic pathway and related gene expression. Citation guidelines: www.kegg.jp/kegg/kegg1.html

The relationship between genes and metabolites can be better understood by simultaneously mapping the DEMs and DEGs of the same group to the KEGG pathway map (Fig. S3). The DEGs and DEMs of R2 vs R6 and W2 vs W6 were primarily concentrated in “photosynthesis,” “arginine biosynthesis,” and “carbon metabolism.” In particular, there were 18 genes annotated in the photosynthetic pathway by R2 vs R6, and 7 genes annotated by W2 vs W6. Furthermore, three enzymes were common among these genes, namely, photosystem II oxygen evolving-enhancer protein 3 (PsbQ), F-type H +—transporting ATPase subunit Delta (delta), and F-type H +—transporting ATPase subunit A (ATPB). PsbQ and delta were significantly downregulated in R2 vs R6 compared to that in W2 vs W6. In R2 vs R6, gene-loc110706344 and gene-loc110693140 act on the PsbQ enzyme; gene-loc110711486 acts on the delta enzyme, gene-loc110711486 and novel 2685 acts on ATPB enzyme Meanwhile, in W2 vs W6, gene-loc110693453 acts on PsbQ enzyme, whereas novel 8867 gene acts on delta and ATPB enzymes. In general, the expression of these genes and that of key metabolites were higher in R2 vs R6 than in W2 vs W6 (Fig. 7; https://www.kegg.jp/pathway/ko00195. Kanehisa laboratory provided permission [29]).

In R2 vs R6, R2 vs R7, and R6 vs R7, DEGs were also enriched in the “arginine biosynthesis pathway.” Furthermore, the three groups shared three key metabolites in this pathway, namely, N-acetylcholine, citrulline, and arginine, which were downregulated in R2 vs R7 and R6 vs R7 and upregulated in R2 vs R6.

### Chlorophyll performance under different potassium levels

**Fig. 8:**
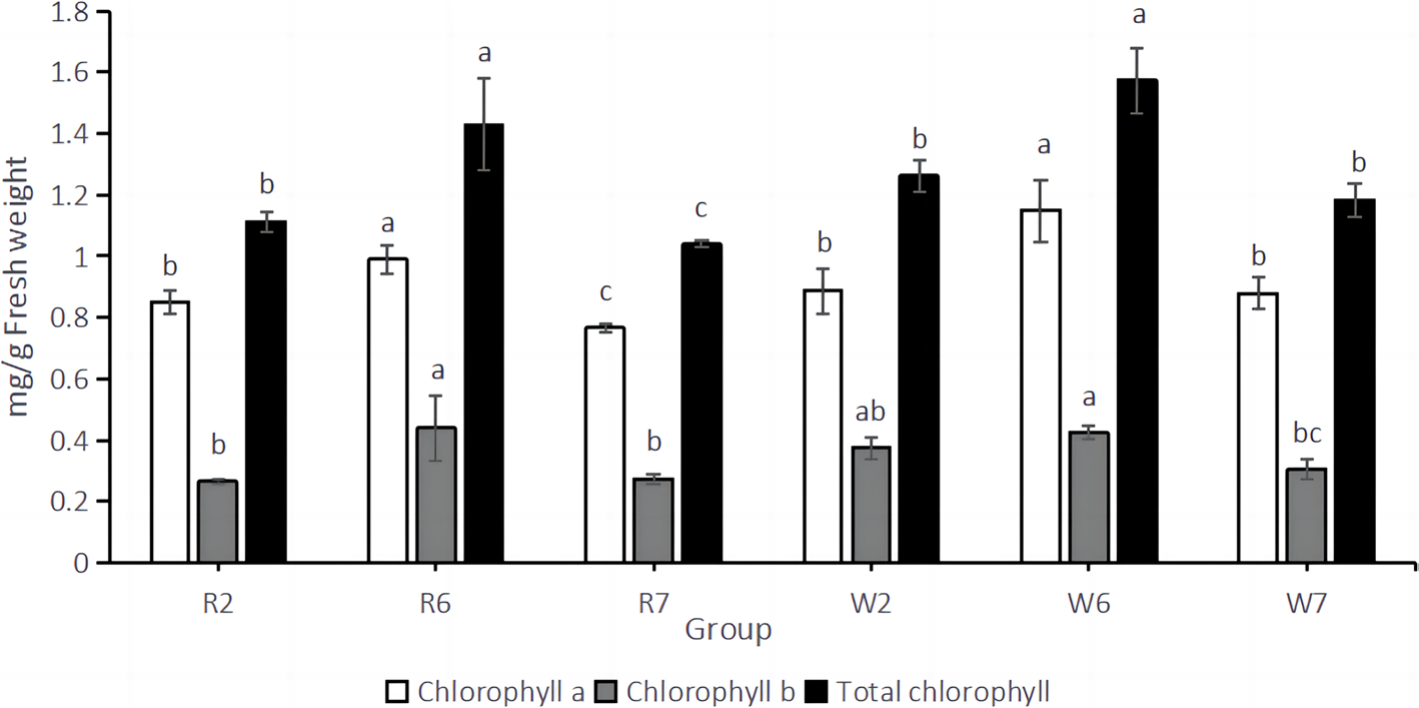
Chlorophyll content

Through the comparison of the six treatments (Fig. 8), it can be seen that in *chlorophyll a*, the content of R6 was significantly higher than R2 and R7, and the lowest in R7; The content of *chlorophyll a* in W6 was significantly higher than that in W2 and W7, and the lowest was W7. In chlorophyll b, the content of *chlorophyll b* in R6 was significantly higher than that in R2 and R7, and there was no significant difference between R2 and R7 groups; the content of *chlorophyll b* in W6 was the highest and significantly higher than that in W7, but the difference between W6 and W2 was not significant. The total chlorophyll content of R6 was significantly higher than that of R2 and R7, and the lowest was R7. The total chlorophyll content of W6 was significantly higher than that of W2 and W7, and the lowest was W7. Through the detection of chlorophyll content, we found that the expression trends of chlorophyll a, chlorophyll b, and total chlorophyll of different varieties were consistent under the same treatment, and the chlorophyll content would be significantly reduced in the case of potassium deficiency.

### RT-qPCR

**Fig. 9:**
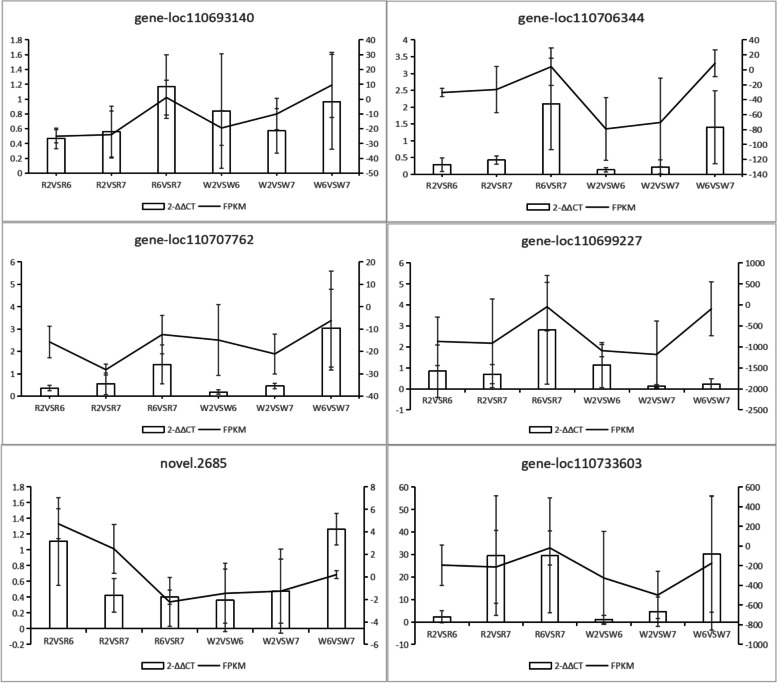
RT-qPCR diagram

To determine the authenticity and reliability of transcriptome data and extent of differential candidate gene expression, RT-qPCR verification was performed on several key genes. *Tub1* was selected as the internal reference gene. Table S2 presents the internal reference gene, as well as the ten verified genes and their primer pairs. The RT-qPCR results of six genes (gene-loc110693140, gene-loc110706344, gene-loc110707762, gene-loc110699227, novel.2685, and gene-loc110733603), among the ten verified genes, were consistent with RNA SEP data, confirming the reliability of transcriptome data obtained in this study (Fig. 9).

## Discussion

### Effect of potassium on the photosynthetic pathway in quinoa seedlings

In the current study, the DEMs and DEGs identified in the potassium-deficiency treatment and control treatment groups were significantly enriched in the photosynthetic pathway. Moreover, we show that the photosynthetic system of quinoa is impacted by potassium deficiency; inhibition of this system leads to a significant decrease in the accumulation of organic matter, which is not conducive to normal plant growth, and may partially account the dwarfism of potassium-deficient crops. Meanwhile, Naciri et al. [30] showed that potassium supplementation improves photosynthesis and promotes chlorophyll synthesis in hydroponic tomato. Similarly, Yu et al. [31] consistently showed that an appropriate K concentration is required for the formation of photosynthetic pigments, non-enzymatic and enzymatic antioxidants, metabolic processes, and nutrient absorption. Song et al. [32] showed that external K supply reduces zinc toxicity on peach seedlings, while enhancing photosynthesis and the antioxidant plant defense system. In contrast, Fontana et al. [33] showed that K deficiency significantly affects photosynthesis and respiration in cotton seedlings, consequently inhibiting their growth and development. Hence, these studies indicate that a major physiological effect of K deficiency is inhibition of net CO_2_ assimilation during photosynthesis [34], which is relatively consistent with the findings of the current study. The occurrence of photosynthesis was directly related to chlorophyll. When K was sufficient, it could promote the synthesis of chlorophyll, thereby improving plant photosynthesis and promoting plant development. In our study, the chlorophyll content of plants in the K deficiency state was the highest. We speculated that it may be due to the influence of K deficiency stress, which produces some negative feedback effect and accelerates the synthesis of their own chlorophyll, so as to maintain the smooth progress of photosynthesis and maintain the normal growth of plants.

In this study, red and white quinoa were grown under three different K fertilization conditions, among which, R2 vs R7 and W2 vs W7 showed no common significant pathway differences; hence, our analysis focused on R2 vs R6 and W2 vs W6, the metabolome and transcriptome results for which revealed enrichment of the photosynthetic pathway as the most significant. In particular, R2 vs R6 and W2 vs W6 shared three common enzymes in the photosynthetic pathway, namely, PsbQ, delta, and atpB, which we speculated to be key enzymes affecting photosynthetic carbon fixation under potassium deficiency. PsbQ is a photosystem II enzyme, while both delta and atpB are components of ATP synthase. PsbQ and the delta subunit were significantly downregulated in the R2 vs R6 analysis compared with W2 vs W6. The function of photosystem II is to use the absorbed light energy to split a water molecule and transfer the released electron to plastid quinone through the electron transport chain. Concomitantly, a proton (H^+^) gradient is established on either side of the thylakoid membrane through water oxidation and PQB2^−^ reduction. ATP synthase, also known as FoF1 ATPase, then catalyzes the synthesis of energy-rich ATP. During respiration or photosynthesis, the energy released through the electron transport chain is first converted into the transmembrane proton (H +) ladder, along which the proton flows. ADP + PI can synthesize ATP through ATP synthase [35]. It is postulated that the PsbQ enzyme on photosystem II and the delta subunit of ATP synthetase are significantly downregulated in quinoa seedlings under potassium deficiency; a subsequent series of chain reactions impact the normal process, thereby reducing photosynthesis efficiency and affecting the normal growth and development of seedlings.

### Effect of potassium on arginine biosynthesis in quinoa seedlings

In this study, R2 vs R6, R2 vs R7, and R6 vs R7 showed a common metabolic pathway, i.e., arginine biosynthesis. By comparing the pathway metabolic maps of the three groups, we found that they contained three key metabolites: N-Acetylcholine, citrulline, and arginine. Arginine is the amino acid with the highest (4/6) nitrogen to carbon (N/C) ratio and performs the most functions in plants. In fact, it is not only a component of proteins and a medium for N storage and transport but also an osmotic regulator. Additionally, arginine participates in proline and NO synthesis through the urea cycle and citrulline NO cycle [36].

Arginine synthesis and its regulation have been well studied in prokaryotes, fungi, and animals [37–39]; in contrast, corresponding data in plants are scarce, with the exception of a few studies largely focused on Arabidopsis [40, 41]. These studies have reported that arginine can improve the ability of crops to resist salt stress, help to enhance root development, and is the precursor of polyamine synthesis of endogenous hormones in plants. However, proline, which is closely related to arginine metabolism, also has an important role in plant stress resistance. Indeed, during arginine and proline metabolism, arginine can be converted into proline to improve the stress response [42–45].

N-Acetylcholine is an intermediate in the enzymatic synthesis of L-arginine [46]. Citrulline is an α-amino acid produced from ornithine and amino formyl phosphate in the urea cycle; alternatively, it may be a by-product of NO synthesis from arginine catalyzed by nitric oxide synthase. Arginine participates in the ornithine cycle in the human body, where it converts ammonia into non-toxic urea through the ornithine cycle, which is discharged through urine to reduce the ammonia concentration in the blood. Moreover, arginine has a high concentration of hydrogen ions, which can help correct the acid–base imbalance in hepatic encephalopathy [47]. Together with histidine and lysine, arginine is a basic amino acid, all of which were downregulated in R2 vs R7 and R6 vs R7 comparisons but upregulated in R2 vs R6. We speculate that potassium deficiency in quinoa seedlings promotes arginine biosynthesis in an effort to improve the adaptability of quinoa seedlings.  

## Conclusions

In this study, we discussed a response mechanism of quinoa seedlings under potassium deficiency, the primary components of which included photosynthesis and arginine biosynthetic pathway. When quinoa seedlings are deficient in potassium, the PsbQ enzyme in photosystem II and delta subunit of ATP synthase become significantly downregulated; the subsequent series of chain reactions reduce the photosynthetic efficiency. In addition, the identified differential genes and metabolites are significantly enriched in the arginine biosynthetic pathway; arginine and its derivatives have important roles in the resistance of quinoa to low potassium stress. Therefore, we speculate that altering the photosynthesis pathway is a passive response of quinoa seedlings when exposed to low potassium stress, whereas enriching arginine and its derivatives is an active response to these conditions.

Collectively, the findings of this study provide novel insights into the molecular mechanisms initiated by quinoa seedlings in potassium-deficient and normal conditions, thereby, describing a new pathway by which quinoa establishes resistance to low potassium stress. 

### Experimental procedures

#### Material selection

**Fig. 10:**
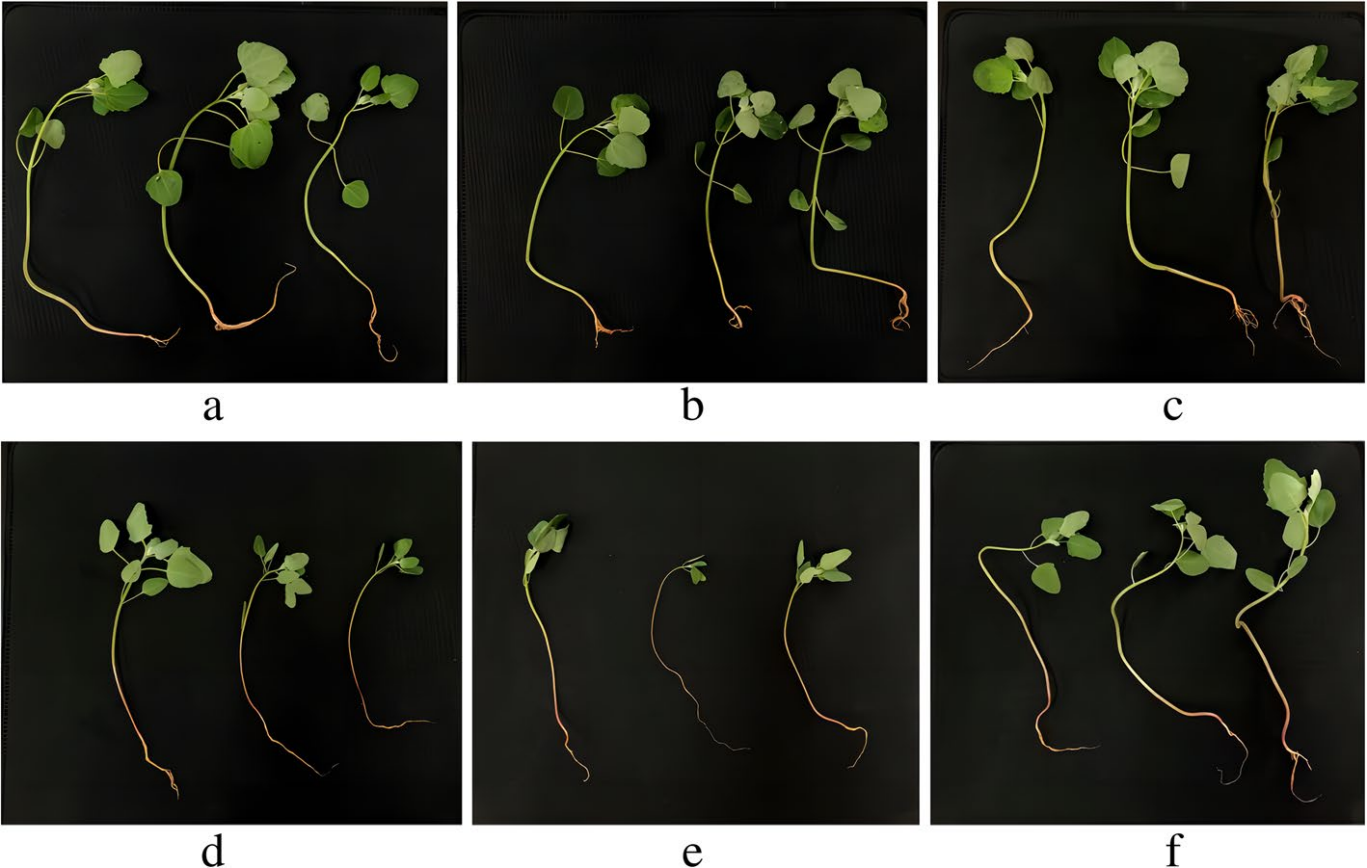
Experimental materials. **a**) R2; **b**) R6; **c**) R7; **d**) W2; **e**) W6; **f**) W7

Two high-generation cultivars of red (dianli-1299) and white Chenopodium (dianli-71) independently bred by Yunnan Agricultural University were planted in the modern Agricultural Education and Scientific Research Base of Yunnan Agricultural University in Xundian County, Kunming (25° 20’ N; 102° 41’ E). Uniform grains were selected and evenly planted in pots (117 cm × 39 cm × 65 cm). The K_2_O treatment rates applied to two lines were 0 (R6, W6), 112.5 (R2, W2), and 337.5 kg ha^−1^ (R7, W7). The P_2_O_5_ and N fertilization rates for all K treatments were both 112.5 kg ha^−2^ with three repetitions. Conventional water management was used during the experimental period (average temperature: 25.6 ℃; sunshine duration: approximately 10 h; sowing depth: 2–3 cm; CH_4_N_2_O, P_2_O_5_, and K_2_O contents in the growth medium were 2.75 1.66, and 1.18 g kg^−1^, respectively). Fertilization was initiated when seedlings reached the second-leaf stage. The aboveground portions of quinoa seedlings were randomly sampled 30 days after fertilizer application (Fig. 10); the samples were immediately frozen in liquid nitrogen and stored at -80 ℃. Metabolome determination and transcriptome sequencing validation were performed in Wuhan Metwell Biotechnology Co., Ltd. (www.metalware).

### Metabolome detection and analysis

#### Metabolite extraction

The biological samples were placed in a freeze dryer (Scientz-100F) for vacuum freeze-drying, and the samples were ground (30 Hz, 1.5 min) to a powder with a grinder (400 mm, Retsch). For analysis, 100 mg of sample powder was weighed and dissolved in 1.2 ml of 70% methanol for extraction. They were then vortexed once every 30 min for 30 s, six times in total, and placed at 4 °C overnight. After centrifugation for 10 min at 12,000 rpm, the supernatant was absorbed and filtered through microporous filter membranes (0.22-μM pore size) prior to storing in injection bottles for UPLC-MS/MS analysis.

The sample extracts were analyzed using an UPLC-ESI–MS/MS system (UPLC, SHIMADZU Nexera X2, www.shimadzu.com.cn/; MS, Applied Biosystems 4500 Q TRAP, www.appliedbiosystems.com.cn/). The analytical conditions were as follows: UPLC: column, Agilent SB-C18 (1.8 µm, 2.1 mm*100 mm). The mobile phase comprised solvent A, pure water with 0.1% formic acid, and solvent B, acetonitrile with 0.1% formic acid. Sample measurements were performed with a gradient program that employed the starting conditions of 95% A, 5% B. Within 9 min, a linear gradient to 5% A, 95% B was programmed, and maintained for 1 min. Subsequently, a composition of 95% A, 5.0% B was adjusted within 1.10 min and maintained for 2.9 min. The flow velocity was set as 0.35 ml/min. The column oven was set to 40 °C, and the injection volume was 4 μl. The effluent was alternatively connected to an ESI-triple quadrupole-linear ion trap (QTRAP)-MS.

#### Database search

The raw data file generated using UPLC-MS/MS was processed with Compound Discoverer 3.1 (cd3.1, Thermo Fisher); the peaks represent the collection and quantification of each metabolite. Retention time tolerance, actual quality tolerance, signal strength tolerance, signal-to-noise ratio, and minimum strength were 0.2 min, 5 ppm, 30%, 3, and 100,000, respectively. Peak intensities were normalized to the total spectral intensity. Based on the molecular formula prediction of additive ions, the ion and fragment ion peaks of normalized data were used, and matched with mzCloud (https://www.mzcloud.org/), mzVault, and MassList databases to obtain accurate qualitative and relative quantitative results. Python (Python version 2.7.6), R statistical software (r version r-3.4.3), and CentOS (CentOS release 6.6) were used for statistical analysis. When the data were not normally distributed, an area-normalization method was applied.

#### Data analysis

Metabolites were annotated using the KEGG database (http://www.genome.jp/kegg/), HMDB database (http://www.hmdb.ca/), and LipidMaps database (http://www.lipidmaps.org/). Metax is a flexible and comprehensive metabolomics data processing software that can support PCA and partial least-squares discriminant analysis (PLS-DA). Statistical significance (*p*-value) was calculated by univariate analysis (*t* test). Metabolites with VIP > 1, *p* < 0.05, fold change (FC) ≥ 2 or ≤ 0.5 were selected as DEMs. Pearson’s correlation coefficients were calculated among all metabolites to identify relationships among all metabolites.

#### Transcriptome sequencing and analysis

The experimental process of transcriptome sequencing includes RNA extraction and detection. RNA degradation and contamination was monitored on 1% agarose gels. RNA purity was checked using the Nanophotometer® spectrophotometer (IMPLEN, CA, USA). RNA concentration was measured using Qubit® RNA Assay Kit in Qubit® 2.0 Fluorometer (Life Technologies, CA, USA). RNA integrity was assessed using the RNA Nano 6000 Assay Kit of the Bioanalyzer 2100 system (Agilent Technologies, CA, USA).

The library was prepared for transcriptome sequencing by first employing fastp (v0.19.3) to filter the original data and remove reads with adapters. When the N content in any sequencing read exceeded 10% of the base number of reads or when the low-quality (Q ≤ 20) base number contained in any sequencing read exceeded 50% of the total base read number, the paired reads were removed to obtain clean reads. Next, the reference genome and its annotation files were downloaded from the designated website, the index was constructed using HISAT (v2.1.0), and clean reads were aligned to the reference genome. StringTie (v1.3.4d) was used to predict new genes. StringTie applied a network flow algorithm and optional de novo assembly to splice transcripts. Additionally, featureCounts (v1.6.2) was employed to calculate the gene alignment and the FPKM of each gene according to the gene length. Differential expression analysis between two groups was performed using DESeq2 (v1.22.1), and p-values were corrected using the Benjamini & Hochberg method. The corrected p-value and | log2 foldchange | were established as the thresholds for significant differential expression.

For KEGG enrichment analysis, hypergeometric distribution testing was performed for the pathway unit. For GO, the analysis is based on GO terms.

#### Combined analysis of transcriptome and metabolome profiles

The batch data from the transcriptome and metabolome analyses were normalized and statistically analyzed to establish the relationship between molecules at different levels. Using combined biological function analysis, metabolic pathway enrichment, and correlation analysis, we systematically analyzed the biological molecular functions and corresponding regulatory mechanisms to develop a comprehensive understanding of the general trend and direction of biological variation. Finally, we proposed a model of the mechanism accounting for molecular biological variation and selected key metabolic pathways or genes and metabolites for subsequent in-depth experimental analysis and application.

#### RT-qPCR

RNA was extracted from the aboveground portion of treated quinoa seedlings for RT-qPCR. The specific primers for the 15 genes were designed by Beacon Designer 7.9. RT-qPCR was performed on a 96-well StepOnePlus instrument (Applied Biosystems, CA, USA) using PerfectStart™ SYBR qPCR SuperMix (TransGen Biotech, Beijing, China), according to manufacturer’s instructions. The reaction system comprised a 20 µL volume including 10 µl of 2 × PerfectStart™ SYBR qPCR SuperMix, 0.4 µL of calibration solution, 5.8 µL of nuclease-free water, 0.4 µL of each primer (10 mm), and 3 μL (200 μg/μL) of cDNA. The thermal cycle was as follows: 94 °C (30 s), 94 °C (5 s) 40 cycles, 60 °C 30 s. The *Tub1* gene was used as the internal control gene, and the relative transcription level was calculated using the 2^−∆∆CT^ method.

## Supplementary Information

**Additional file 1:** **Figure S1.** a,b QC sample mass spectrometry TIC overlap diagram. **Figure S2 **kmeans cluster analysis diagram. **Figure S3.** KEGG channel histogram a) R2 vs R6; b) W2 vs W6; c) R2 vs R7; d) W2 vs W7.** Table S1**. Analysis of metabolites. **Table S2. **Pecific primer pairs of selected genes.

## Data Availability

The datasets generated and/or analyzed during the current study are available in the [NCBI] (National Center for Biotechnology Information) repository, [PRJNA839110]. We hereby declare that the materials used in this study (dianli-1299, dianli-71) were independently selected and bred by Qin Peng’s group at Yunnan Agricultural University and have the right to use them.
