# Supplementary material for: Metabolome and transcriptome profiles in quinoa seedlings in response to potassium supply
Source: BMC Plant Biol. 2022 Dec 21;22:604. doi: 10.1186/s12870-022-03928-8 (PMC9768898; doi:10.1186/s12870-022-03928-8)
Supplement: Supplementary file 1 — Additional file 1: Figure S1. a,b QC sample mass spectrometry TIC overlap diagram. Figure S2 kmeans cluster analysis diagram. Figure S3. KEGG channel histogram a) R2 vs R6; b) W2 vs W6; c) R2 vs R7; d) W2 vs W7. Table S1. Analysis of metabolites. Table S2. Pecific primer pairs of selected genes. [file 12870_2022_3928_MOESM1_ESM.docx]

Additonal file 1


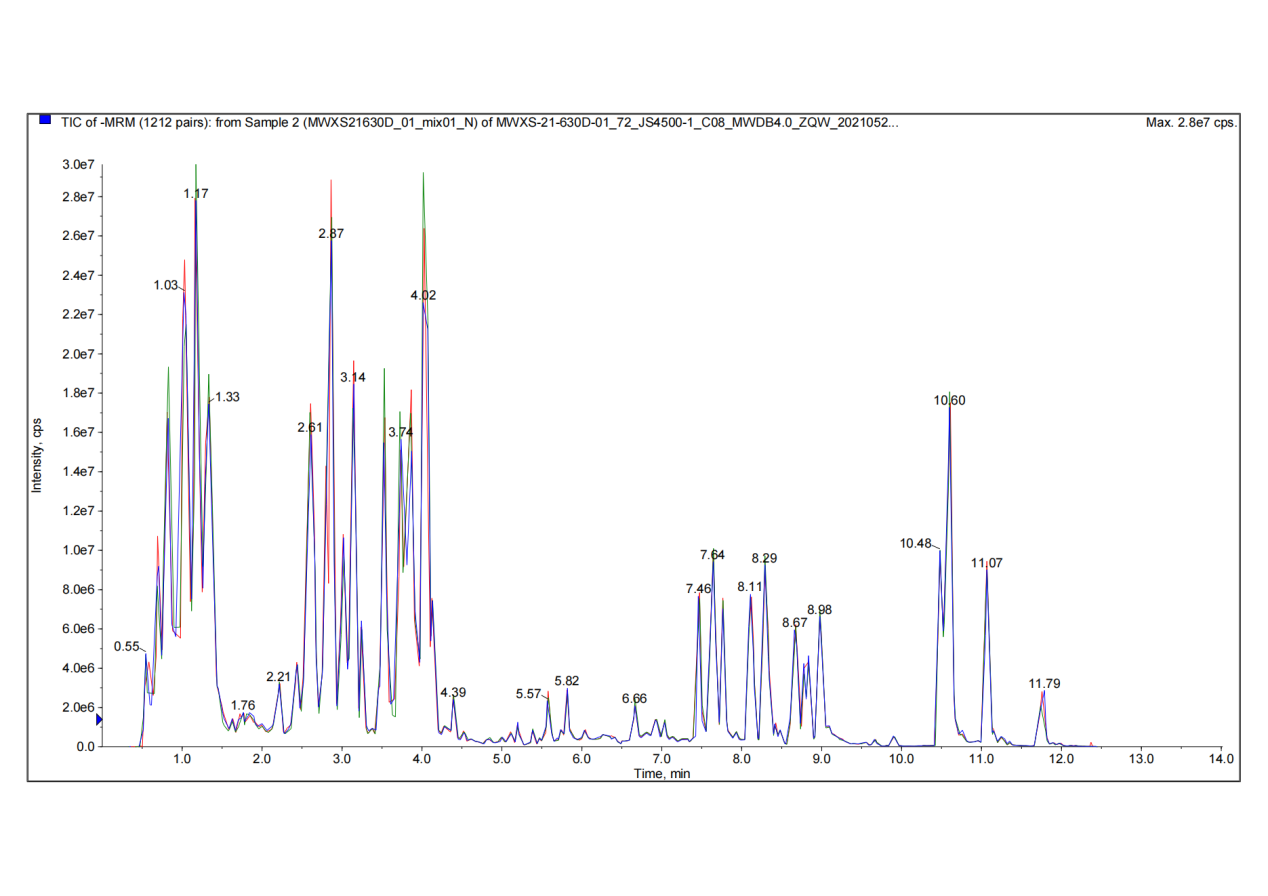


a


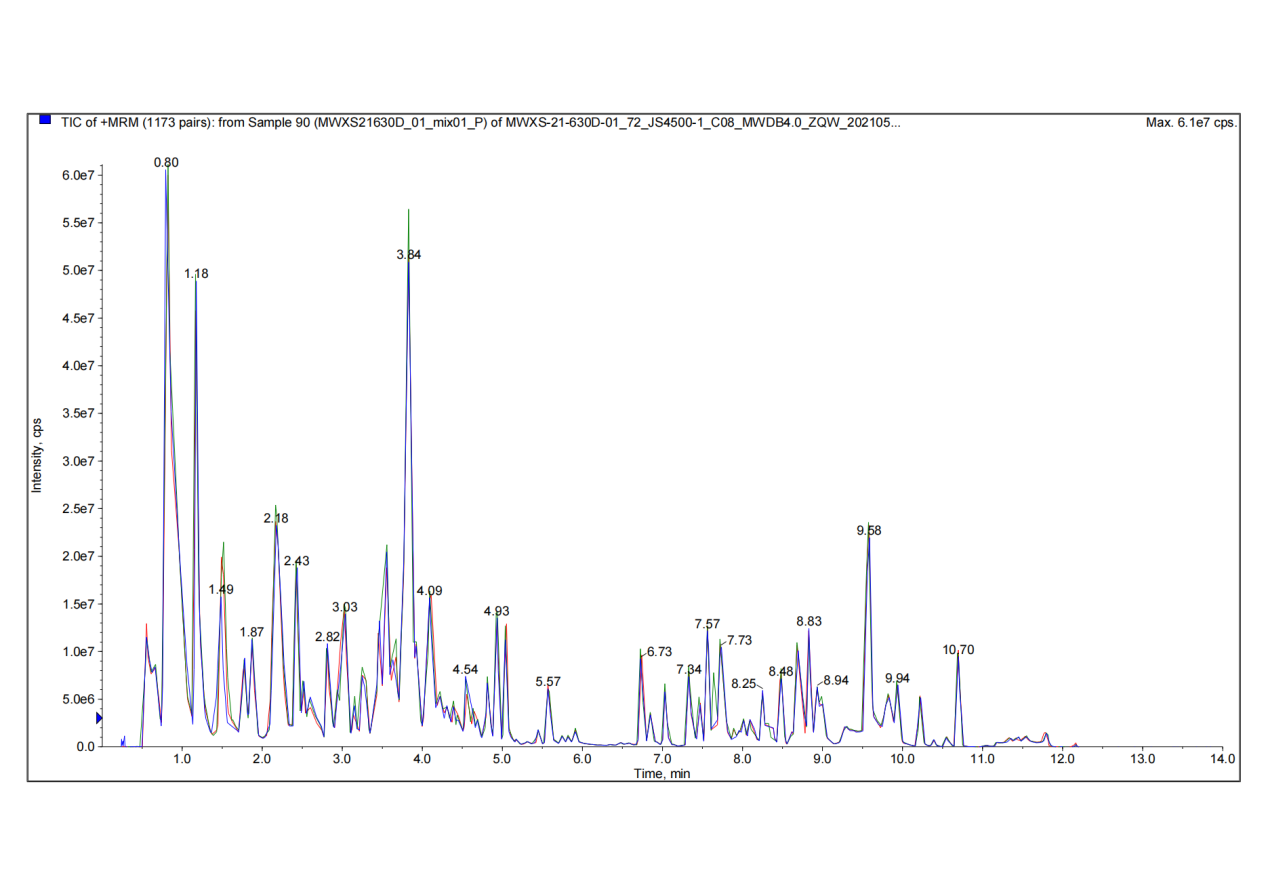
b

**Figure S1.** a,b QC sample mass spectrometry TIC overlap diagram;


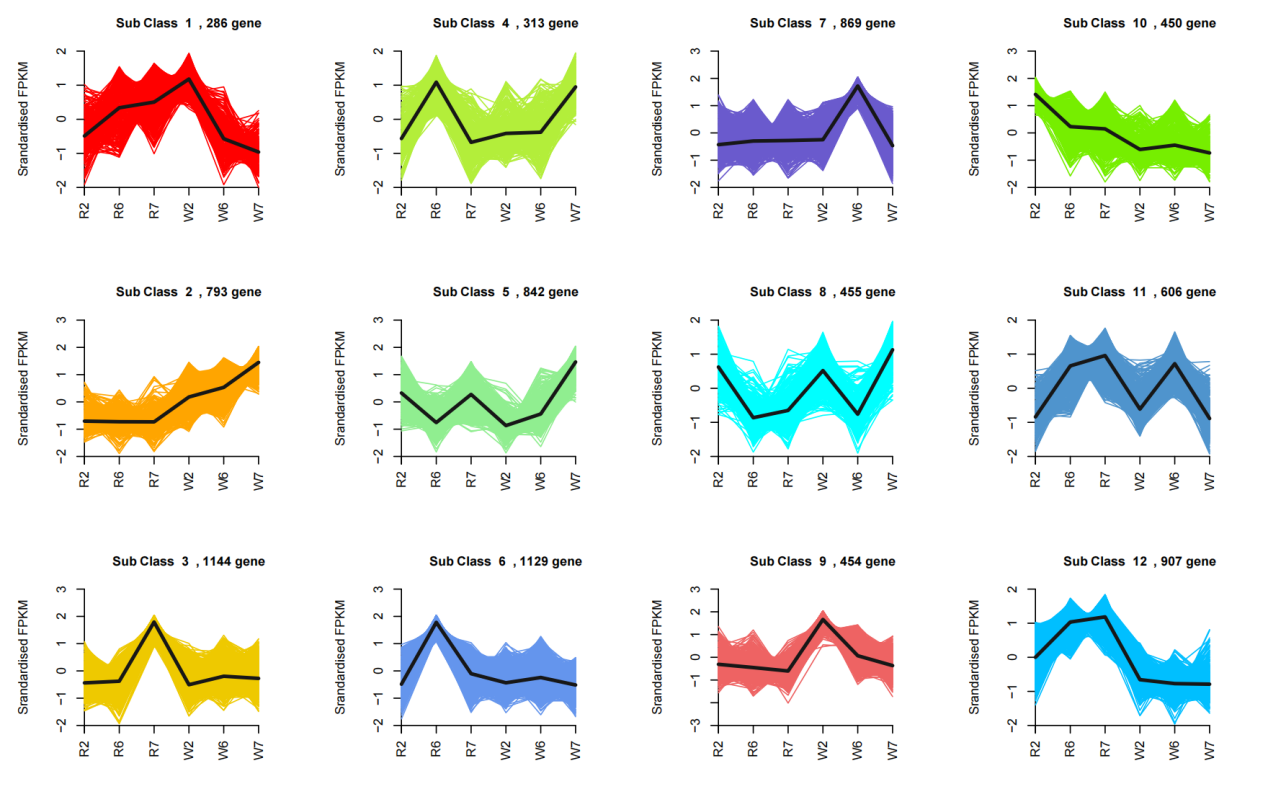


**Figure S2** kmeans cluster analysis diagram


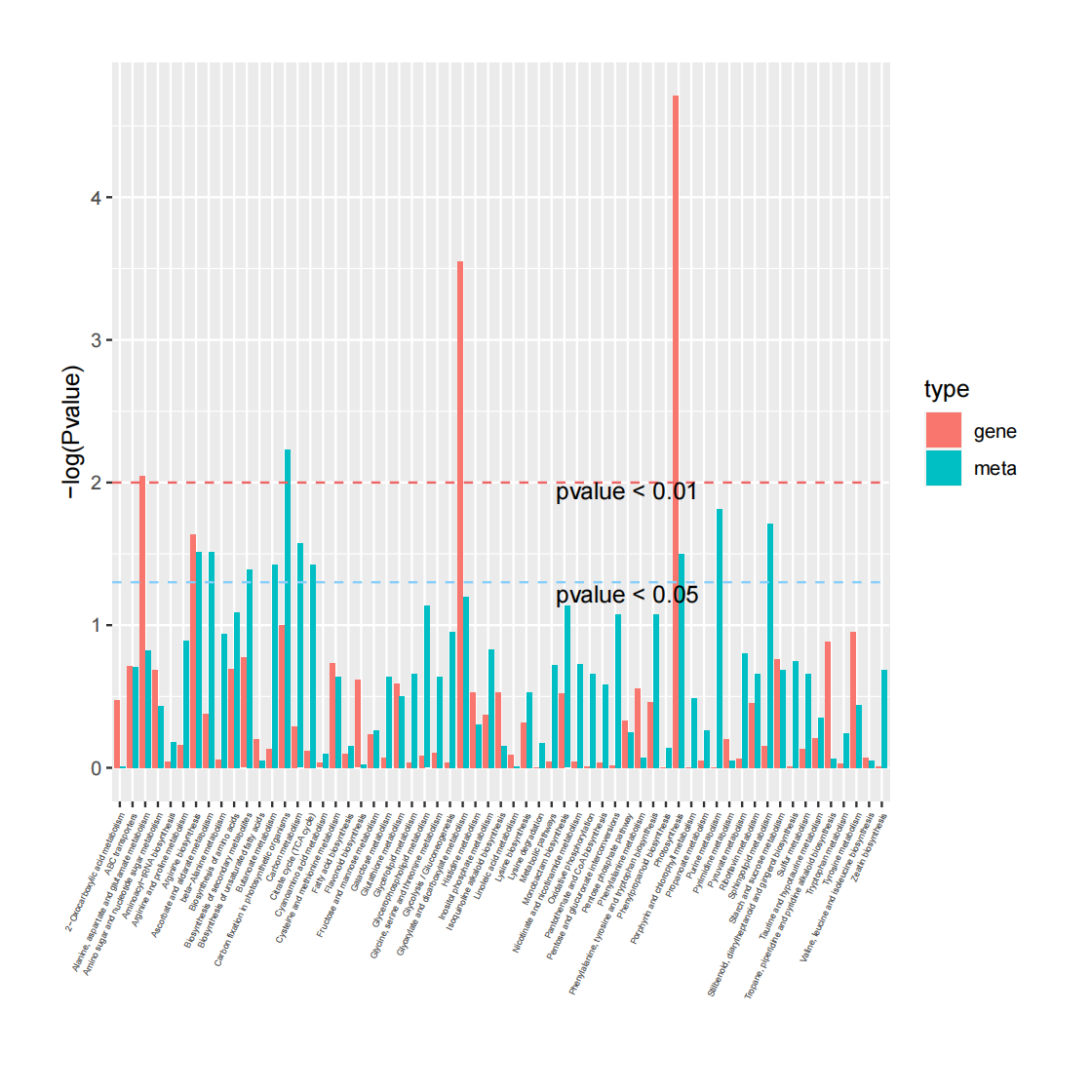


a


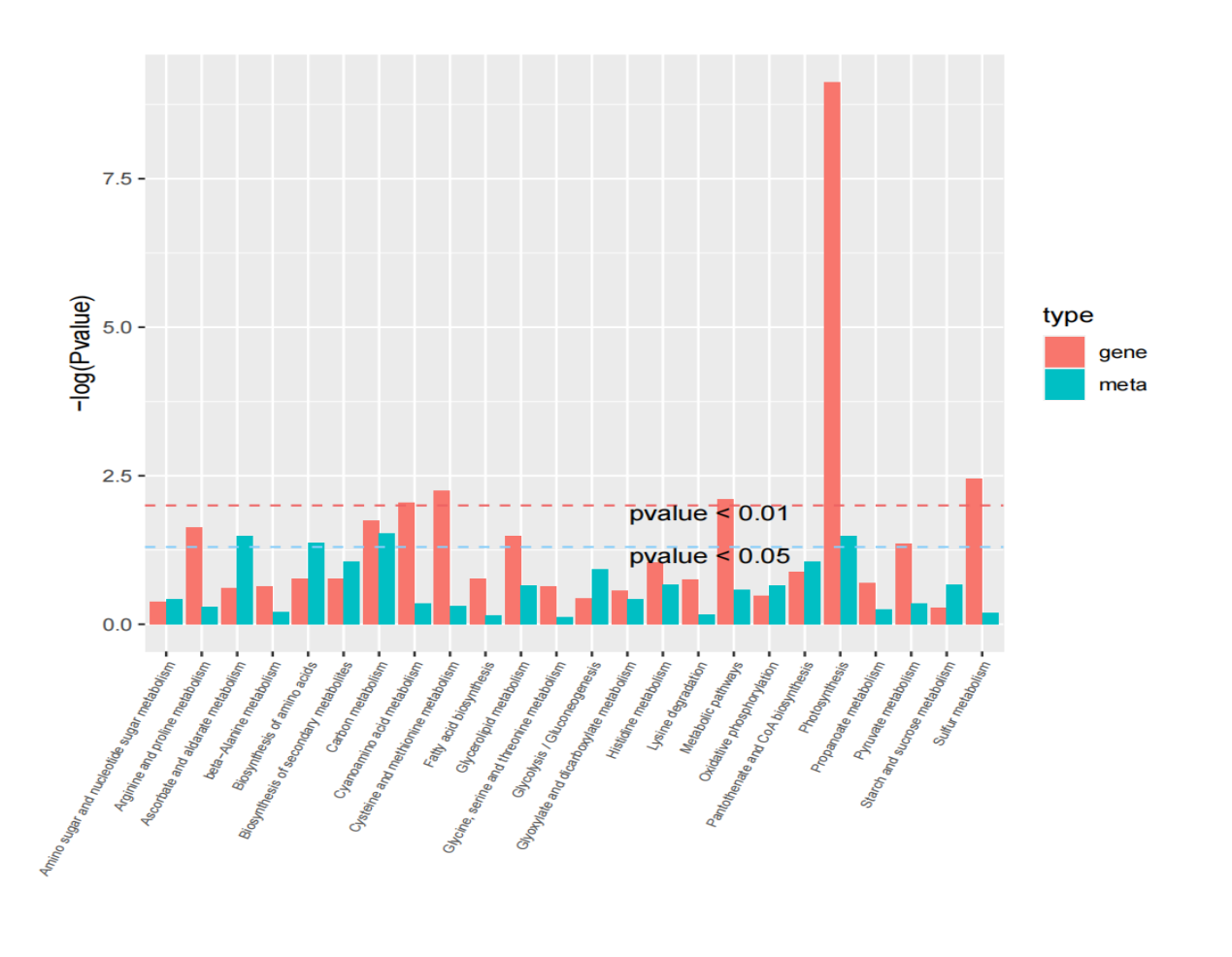


b


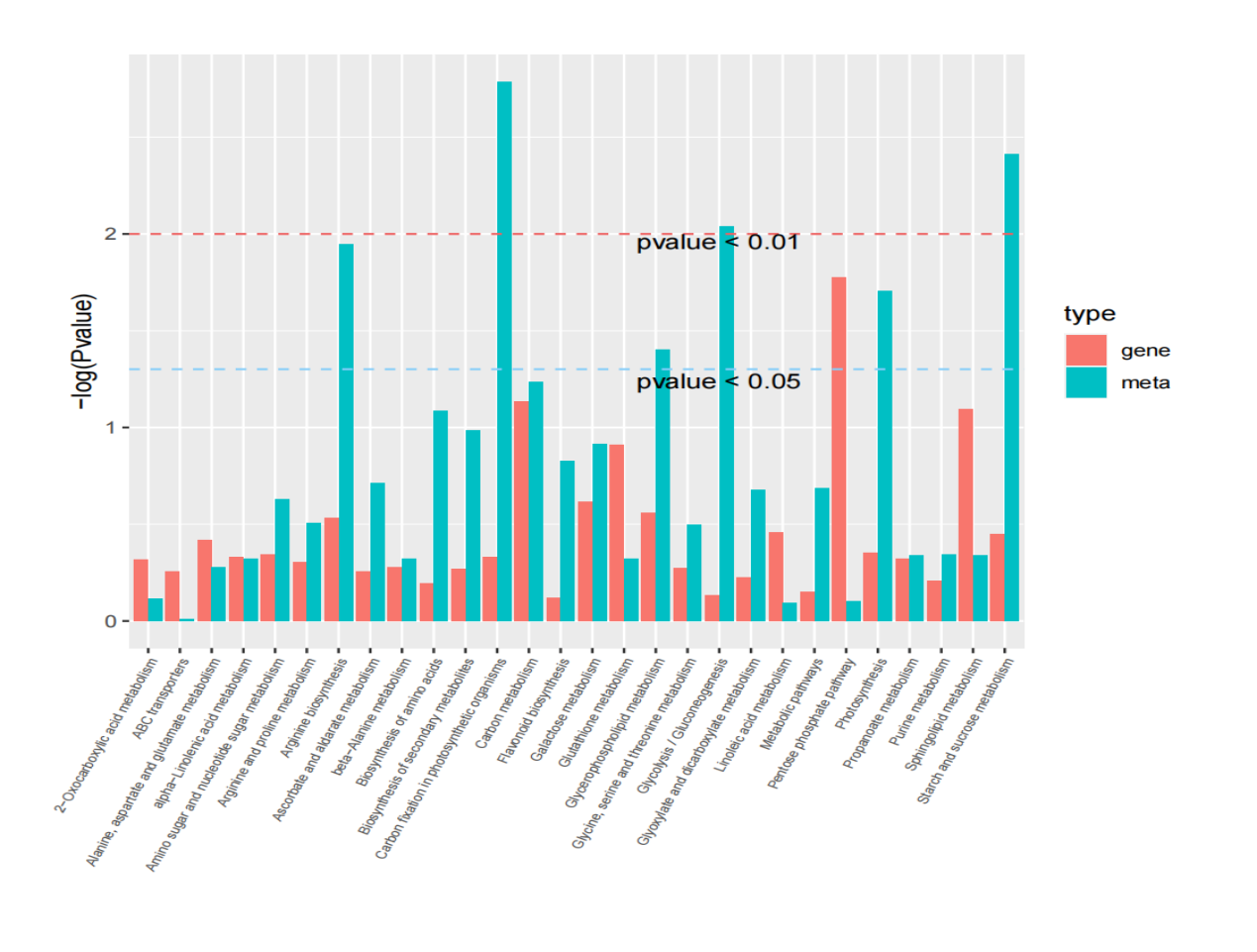


c


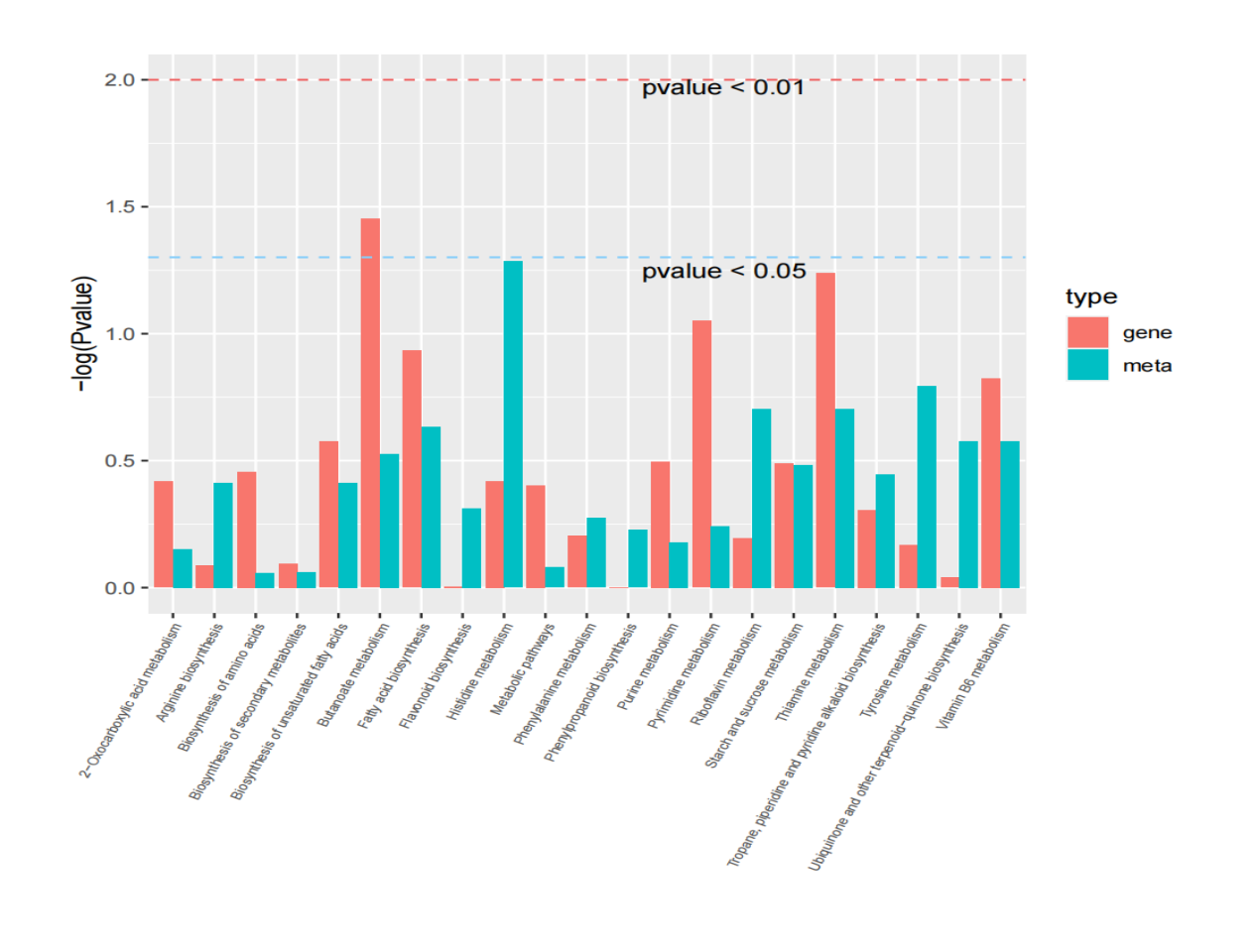


d

**Figure S3.** KEGG channel histogram a) R2 vs R6; b) W2 vs W6; c) R2 vs R7; d) W2 vs W7.

**Table S1**

| **Species of metabolites** | **Number of metabolites** |
| --- | --- |
| Amino acids and their derivatives | 98 |
| Phenolic acids | 166 |
| Nucleotides and their derivatives | 69 |
| Flavones | 172 |
| Lignans and coumarins | 24 |
| Blending quality | 9 |
| Alkaloids | 78 |
| Terpenoids | 39 |
| Organic acids | 94 |
| Lipids | 177 |
| Quinones | 11 |
| Other | 120 |
| Total | 1057 |

Analysis of metabolites.

**Table S2**

| Gene-ID | Primer | 5^,^ to 3^,^ |
| --- | --- | --- |
| TUB1(Internal reference gene) | Forward Primer  Reverse Primer | ATTGAGCGTCCTACCTACACTA  TGGTCTGGAACTCATTCACATC |
| gene-LOC110693140 | Forward Primer  Reverse Primer | AACCGACTCTTCAACAAT  AATCTATCCATCACCTCTTG |
| gene-LOC110706344 | Forward Primer  Reverse Primer | GGCTAGGATTAAGAATACA  TCAGGAACCATACTCATA |
| gene-LOC110711486 | Forward Primer  Reverse Primer | CTCATACGGCTAACTTCCT  GTCACCACTGCTACTTCA |
| gene-LOC110707762 | Forward Primer  Reverse Primer | GTAGTGTAGACCAATCAG  ACTAATAGGATAAGCAACA |
| gene-LOC110699227 | Forward Primer  Reverse Primer | CTTAGATGATGACCAGATAGC  CTCCTCCTTGTGAGTCTC |
| novel.2685 | Forward Primer  Reverse Primer | CAACTACTAATTCATCGGCTAAT  AATACATTCAACCAACTCCAAT |
| gene-LOC110694878 | Forward Primer  Reverse Primer | CAAGCACCAGGAGAAGAC  TGTTACCACCACGGAATG |
| gene-LOC110722428 | Forward Primer  Reverse Primer | CTGATAAGGTGATTGCTGAG  GGTCCATTGAGTGTTCTTG |
| gene-LOC110733603 | Forward Primer  Reverse Primer | TGCCTTGAATAAGATGAA  TTAATAGTATCCTCCTTCTG |
| novel.8897 | Forward Primer  Reverse Primer | ATAAGTGTCAGCCGAGTT  TAATGCGTGGATTGAAGAC |

Pecific primer pairs of selected genes
